# Management of Critical Wunderlich Syndrome: A Case Report and Review of Therapeutic Strategies

**DOI:** 10.1155/criu/2129870

**Published:** 2025-03-15

**Authors:** Samer Danaf, Yehya Tlaiss, Mohamad Chams, Georges Assaf, Mohammad Moussa, Imad Ghantous

**Affiliations:** ^1^Department of Urology, University of Balamand, Beirut, Lebanon; ^2^University of Balamand, Beirut, Lebanon; ^3^Department of Urology, Saint Georges Hospital University Medical Center, Beirut, Lebanon; ^4^Faculty of Medicine, Lebanese University, Beirut, Lebanon

**Keywords:** cystic rupture, hematuria, polycystic kidney disease, tranexamic acid, Wunderlich syndrome

## Abstract

This case report presents a compelling instance of Wunderlich syndrome (WS), a rare and nontraumatic medical condition characterized by spontaneous renal hemorrhage into the subcapsular and perirenal spaces. WS poses unique diagnostic and management challenges due to its sudden and substantial hemorrhage presentation that is life-threatening, often accompanied by acute flank pain, hemodynamic instability, and the presence of a flank mass. While the exact pathogenesis remains debated, WS can arise from various renal pathologies, including neoplastic and nonneoplastic conditions. In this case, a 65-year-old male with a complex medical history, including polycystic kidney disease, presented to the emergency room with massive hematuria leading to an abrupt severe drop in hemoglobin levels and hemodynamic instability. Despite aggressive management, including transfusions, the patient ultimately underwent an urgent open left radical nephrectomy due to the severity of the condition and distorted anatomy. Additionally, the report draws attention to the potential use of tranexamic acid in WS cases, stressing the importance of balancing its benefits against associated risks. This case highlights the critical importance of recognizing and promptly addressing WS, highlighting the diverse etiology of this condition, the role of tranexamic acid in controlling bleeding, and the lifesaving role of nephrectomy in cases of significant bleeding and hemodynamic instability.

## 1. Introduction

Wunderlich syndrome (WS) is a rare and intriguing medical condition, primarily because of its spontaneous and nontraumatic nature. This syndrome, characterized by renal hemorrhage into the subcapsular and perirenal spaces, poses a unique challenge in diagnosis and management [1]. WS is marked by sudden and substantial hematuria, and its clinical presentation can be challenging. It is also referred to as atraumatic spontaneous rupture of the kidney (ASRK) which was first documented by Bonet in 1679 but coined as WS after Wunderlich in his 1856 documentation [2]. Patients often present with acute flank pain, hemodynamic instability, and the presence of a flank mass, whereby this triad of symptoms is commonly referred to as Lenk's triad [3]. However, these symptoms can mimic various other acute abdominal conditions [4], making its diagnosis a challenging task.

Approximately 5% of WS cases are referred to as idiopathic [5]. However, there are two prevailing theories that attempt to explain its pathogenesis exist. One theory suggests that this syndrome originates from the gradual enlargement of a cyst within the kidney, eventually leading to its rupture over an arterial vessel [3]. An alternative perspective posits that an artery may, over time, open within an existing cyst, causing it to expand and subsequently rupture. The exact causative mechanism remains a subject of ongoing investigation. It is not known whether expansion with increased intracystic pressure occurs with subsequent tearing of blood vessels, or whether hemorrhage from a blood vessel into the cyst occurs first, with subsequent rupture due to cyst expansion [3].

WS can arise from a diverse range of neoplastic and nonneoplastic renal pathologies, with renal neoplasms, particularly angiomyolipoma, being the most frequent origins [6]. Less common causes include vascular disorders such as polyarteritis nodosa, ruptured renal artery aneurysms, and arteriovenous malformations, as well as conditions like ruptured kidney cysts, infections, kidney stones, and blood clotting disorders [3]. Management options for these cases range from conservative management, embolization, and surgical intervention. In acute onset cases where continuous bleeding leads to low hemoglobin levels, embolization of the renal or segmental artery is the preferred course of action for the majority of patients, followed by surgical intervention [7]. Surgical options include partial or radical nephrectomy, depending on the severity of the condition [7].

To highlight the diagnostic challenges and therapeutic considerations surrounding this condition, we present a case of a 65-year-old male who presented to the emergency room with massive hemorrhage from the penis, eventually leading to an urgent open left radical nephrectomy. This case serves as a reminder of the critical importance of recognizing and promptly addressing this rare entity that is potentially life-threatening, known as WS.

## 2. Case Presentation

A 65-year-old male, a nonsmoker, presented to the emergency room with a sudden onset of massive hemorrhage from his penis that had been ongoing for 30 min. The patient had a significant medical history, including adult-type polycystic kidney disease (PKD), atrial fibrillation, obstructive sleep apnea (managed with CPAP), hypertension, dyslipidemia, benign prostatic hyperplasia (BPH), and a surgical history pertinent to a previous history of left kidney cyst unroofing.

Eight years ago, along with a history of pilonidal cyst excision. The patient is on various medications, most notably Xarelto (rivaroxaban). The patient's clinical presentation was marked by severe lethargy, somnolence, and diaphoresis, accompanied by severe pelvic fullness and suprapubic pain. Notably, the patient did not report any flank pain, fever, or chills, and had no prior history of either microscopic or macroscopic hematuria. Upon examination, the patient's vital signs revealed a blood pressure of 90/50 mmHg, temperature of 36.7°C, and a heart rate of 105 beats per minute. Physical examination indicated suprapubic tenderness without costovertebral angle (CVA) tenderness bilaterally. The genital examination was unremarkable.

Mucous membranes were pale, and capillary refill time was delayed. Upon arrival at the emergency room and recognition of active bleeding, rivaroxaban was discontinued immediately to prevent further hemodynamic compromise.

The initial management goals included close monitoring and patient stabilization. A vigorous intravenous (IV) hydration was initiated, and the patient was attached to a monitor. Continuous bladder irrigation (CBI) using a three-way Foley catheter was attempted, but the outflow was obstructed due to blood clots, necessitating the insertion of a 24-Fr coude tip Foley catheter with initial manual bladder irrigation removing multiple blood clots followed by CBI due to continuous bleeding. Additionally, IV tranexamic acid (Exacyl 1 g IV) was administered to reduce bleeding.

Initial laboratory evaluation revealed significant abnormalities, including a hemoglobin level of 10.3 g/dL compared to 15.6 g/dL 2 years earlier, a hematocrit of 32.1, a platelet count of 538,000, and a white blood cell count of 18.3 × 10^9^/L. Coagulation parameters indicated a prolonged partial thromboplastin time (PTT) of 39.54 and an international normalized ratio (INR) of 1.36. Serum creatinine was measured at 1.16 mg/dL. For clarity and better comparison with standard laboratory values, the patient's results are tabulated below alongside their respective reference ranges (Table 1).

**Figure 1:**
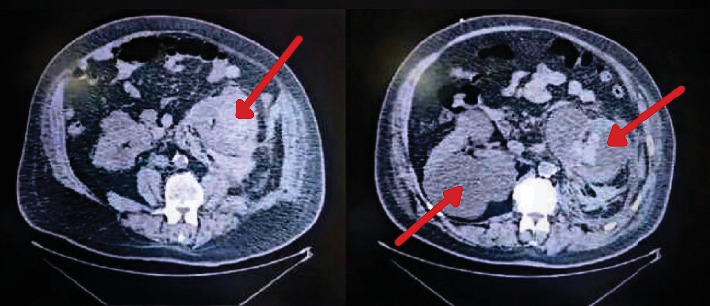
Contrast-enhanced CT scan demonstrating extensive perirenal hematoma associated with Wunderlich syndrome.

Given the persistence of massive hematuria and a drop in blood pressure to 70/40 mmHg despite aggressive IV hydration, central venous access was established, and the patient was started on vasopressors. Over the course of the next few hours, the patient required multiple transfusions of packed red blood cells (PRBC), fresh frozen plasma (FFP), and platelets due to continued bleeding. Despite these measures, hematuria persisted, and patient hemoglobin reached 6.7. Three additional units of PRBC, 2 units of FFP, and 1 unit of platelets were transfused. After initial management, a noncontrast CT scan was performed (Figures 1), which revealed significant findings including a spontaneous nontraumatic left renal cyst hemorrhage and rupture with perirenal and posterior pararenal blood extending along the psoas muscle and common iliac vessels reaching the pelvis. Blood was also seen in the collecting system, ureter, urinary bladder, and upper anterior aspect of the liver. Given the patient's hemodynamic instability, rapidly increasing hematoma size, ongoing bleeding, and poor functional reserve of the affected kidney, a multidisciplinary decision was made to proceed with nephrectomy instead of angioembolization. This decision was reached after consultation with the radiology team and assessment of the imaging findings.

The patient underwent an urgent open left radical nephrectomy and adrenalectomy due to the severity of the condition and distorted anatomy. During the surgery, an additional unit of PRBC and FFP was administered. Subsequent labs postnephrectomy showed stabilization of hemoglobin (8.5) and creatinine levels (2.85).

Following the surgery, the patient had a smooth postoperative course with stabilized vital signs. Active hematuria resolved, and the drain was removed on postoperative Day 4. Hemoglobin levels stabilized at 11 after a total of 11 units of PRBC, 7 units of FFP, and 2 units of platelet transfusions. Creatinine levels also stabilized at 1.6. On the fourth day postoperation, the patient required cystoscopy and bladder declotting, during which a significant amount of bladder clots was evacuated. Subsequently, the urine became clear, and the drain was removed 1 day after declotting. The patient was discharged home after a 10-day hospital stay from the initial presentation. Pathology report indicated multiple renal benign simple cysts with intrarenal hemorrhage, hematoma, and formation of adjacent Gandy-Gamna nodules. The diagnosis of this case was confirmed as WS. It is important to note that a wide range of neoplastic and nonneoplastic renal pathologies can lead to WS, with renal neoplasms being the most common cause. In cases of hemodynamic instability or massive bleeding, nephrectomy is considered a lifesaving appropriate management. The clinical presentation, diagnostic evaluation, and management of this patient's case highlights the importance of recognizing and promptly addressing this rare and potentially life-threatening condition.

## 3. Discussion

WS, characterized by spontaneous renal hemorrhage, represents a rare and confusing clinical entity. The exact etiology of WS remains a topic of ongoing investigation, with two predominant theories considered: one suggesting that it results from the gradual expansion of a renal cyst leading to rupture, and the other proposing that a vascular event within an existing cyst initiates its expansion and subsequent rupture [3]. The case we presented, with PKD being the most probable cause leading to WS, highlights the diversity of renal pathologies that can lead to WS, including neoplastic and nonneoplastic conditions, with renal neoplasms, especially angiomyolipoma, being the most frequent culprit [6]. As seen in our patient, the clinical presentation of WS can be nonspecific, especially in cases of hematuria where it may be confused with bleeding bladder tumors or other causes or urologic bleeding, making its diagnosis a complex challenge. Importantly, the timely detection and management of WS are essential due to the potential for life-threatening hemorrhage.

PKD is associated with several vascular abnormalities, including intracystic hemorrhage, renal artery aneurysms, and arteriovenous malformations [2]. These vascular changes result from the fragility of cyst walls and surrounding vasculature, predisposing patients to spontaneous bleeding events [6]. Studies have reported that arterial abnormalities such as microaneurysms and weakened vessel integrity in PKD contribute significantly to the risk of spontaneous renal hemorrhage [3, 6]. The interplay between these vascular anomalies and anticoagulation therapy likely exacerbated the hemorrhagic event in our patient.

The primary therapeutic approach for WS relies on the severity of the condition and the patient's hemodynamic stability. In cases of acute onset with continuous bleeding, embolization is typically favored [8], and surgery is another important option, with emphasis on nephron-sparing surgery [9]. Surgical options range from partial to radical nephrectomy, depending on the extent of the bleeding and the underlying renal pathology [7]. Partial nephrectomy, when feasible, is preferred to preserve some renal function [9]. Furthermore, the role of interventional radiology techniques, such as embolization of the renal or segmental artery, may be considered in cases of severe hemorrhage [10]. In our case, an urgent open left radical nephrectomy and adrenalectomy were performed due to the severity of the condition and distorted anatomy, along with continuous transfusions, ultimately stabilizing the patient's vital signs and resolving active hematuria.

In cases of acute severe bleeding which might include massive hematuria such as in our patient's case, the considered interventions include angioembolization or partial or radical nephrectomy [10]. Factors like patient stability, severity of condition, extent, and location of bleed guide the choice of management. Renal angiography often aids with the diagnosis of the underlying cause in select cases and also allows control of active bleeding [10]. In cases of hemodynamic instability or massive bleeding, nephrectomy is considered the lifesaving appropriate management as reported in most cases [6, 8, 11–14]. Nephrectomy is mostly seen in cases of angiomyolipoma [6, 8, 11, 13, 14] and in renal tumors [9] with the size of the cyst or tumor determining if it is partial or radical [9]. Additionally, the literature records radical nephrectomy being performed over a case of ruptured intraparenchymal renal artery aneurysm [15]. In our case, the patient's condition was rapidly deteriorating as was seen on CT (Figure 1), thus nephrectomy was opted for. While angioembolization is a preferred initial approach in many cases of renal hemorrhage, it may be contraindicated in patients with severe hemodynamic instability, large and rapidly increasing hematomas, and poor renal function, as observed in this case.

Additionally, we would like to highlight the significance of supportive measures in the management of WS, especially in the critical phase of stabilization. Aggressive IV hydration, blood transfusions, and the temporary discontinuation of anticoagulation therapy, are essential elements in patient care [16].

The pathogenesis of this case involves a complex interaction between the patient's underlying adult PKD and his use of the factor Xa inhibitor, rivaroxaban. Rivaroxaban, by inhibiting factor Xa, reduces thrombin generation and subsequent clot formation. In patients with PKD, the kidneys are often structurally compromised with cysts that can spontaneously bleed. Under normal circumstances, minor bleeding from these cysts might clot quickly; however, in the presence of rivaroxaban, the impaired clotting can lead to excessive hemorrhage, as observed in this patient's severe presentation. This illustrates the critical need for careful management of anticoagulation therapy in patients with conditions that predispose them to bleeding.

The management of life-threatening hemorrhage associated with WS requires a comprehensive and tailored approach. While antifibrinolytic agents like tranexamic acid are sometimes considered in hemorrhagic scenarios [17], their role in WS remains limited and controversial due to a lack of robust evidence in the literature. In this case, the decision to administer tranexamic acid was made as part of initial stabilization efforts; however, its impact was likely minimal given the severity of bleeding and the rapid progression to hemodynamic instability. This report emphasizes the critical importance of prioritizing definitive interventions, such as nephrectomy, in cases where bleeding and instability reach critical levels.

In summary, this case highlights the importance of recognizing WS, understanding its diverse etiology and clinical presentations. Timely diagnosis and appropriate management, often involving surgical intervention, are crucial for the successful treatment of WS and the prevention of life-threatening hemorrhage.

## 4. Conclusion

In conclusion, WS presents a unique challenge due to its rarity, diverse etiology, and complex clinical manifestations. The case we presented highlights the need for a high index of suspicion when encountering patients with gross hematuria. The exact cause of WS remains a subject of ongoing investigation, with theories revolving around renal cyst expansion and vascular events within cysts. WS can result from various renal pathologies, including neoplastic and nonneoplastic conditions. Successful management depends on timely diagnosis and appropriate intervention, considering the patient's hemodynamic stability and the extent of bleeding. Surgical approaches, such as nephrectomy, play a crucial role as a lifesaving intervention. Additionally, supportive measures, including hydration and blood transfusions, are essential during the critical phase of stabilization. Additionally, more research is needed on the administration of tranexamic acid, which could become essential in cases similar to ours. This case serves as a reminder of the importance of recognizing and promptly addressing WS, a rare entity that can have life-threatening consequences if not managed appropriately.

## Figures and Tables

**Table 1:** The patient's laboratory results with reference ranges.

**Laboratory test**	**Patient's value**	**Reference range**
Hemoglobin	10.3 g/dL	13.5–17.5 g/dL (males)
Hematocrit	32.1%	41%–53% (males)
Platelet count	538,000/mm^3^	150,000–450,000/mm^3^
White blood cell count	18.3 × 10^9^/L	4.0–11.0 × 10^9^/L
Partial thromboplastin time (PTT)	39.54 s	25–35 s
International normalized ratio (INR)	1.36	0.8–1.2
Serum creatinine	1.16 mg/dL	0.6–1.2 mg/dL

## Data Availability

Data and materials supporting the findings are available upon reasonable request to the corresponding author. Access will comply with ethical and privacy considerations.
